# Reconsidering the incremental prognostic value of variant allele frequency-based stratification beyond European LeukemiaNet 2022 in acute myeloid leukemia

**DOI:** 10.1093/oncolo/oyag406

**Published:** 2026-10-12

**Authors:** Yu Fu, Shasha Qiu

**Affiliations:** Department of the First Clinical College, Hubei University of Medicine , No.30 Renmin South Road, Shiyan, Hubei Province 442000, China; Department of Geriatrics, The Second Affiliated Hospital of Hubei University of Science and Technology, Xianning, Hubei Province 437100, China

We read with great interest the article by Zhao et al., entitled “Clinical implications of variant allele frequencies (VAFs) of genes in patients with acute myeloid leukemia,” recently published in *The Oncologist.*^1^ The authors investigated a clinically relevant question regarding whether incorporating VAFs of recurrently mutated genes could improve risk stratification beyond the European LeukemiaNet (ELN) 2022 recommendations.^2^ They found that incorporating VAF thresholds into risk stratification improved prognostic discrimination compared with ELN 2022 classification, supporting the potential value of VAF-based approaches for refining AML risk assessment.

However, several methodological issues warrant further consideration. Specifically, the reported superiority of the VAF-based model over ELN 2022 may partly reflect optimism bias arising from data-driven threshold selection and model overfitting rather than a robust enhancement in risk discrimination.

First, the reproducibility of the proposed VAF thresholds remains uncertain. Within the same cohort, the optimal VAF thresholds varied between analyses based on overall survival (OS) and progression-free survival (PFS). For example, the optimal thresholds for FLT3-ITD (35% vs 26%), TP53 (10% vs 15%), DNMT3A (45% vs 43%), and CEBPA (2% vs 3%) differed between OS and PFS analyses. Such inconsistency raises concern that these thresholds may represent cohort-specific statistical optima rather than biologically meaningful and reproducible boundaries.

Second, several prognostic associations appear to be influenced by very small patient subsets. The SF3B1 VAF-high subgroup included only six patients but yielded a hazard ratio of 6.35 with a wide confidence interval (1.91-21.11). Similar concerns apply to other high-VAF subgroups, including FLT3-ITD, TP53, and DNMT3A R882, which consisted of only 11–13 patients.

Most importantly, the generalizability of the proposed VAF-based model requires further confirmation. Prediction models should be developed and validated according to transparent reporting principles and appropriate validation strategies.^3^ Although the model demonstrated improved discrimination compared with ELN 2022 in the derivation cohort (C-index, 0.70 vs 0.55), the improvement was attenuated during external validation (C-index, 0.63 vs 0.66). Differences in sequencing platforms between derivation and validation cohorts may further challenge the transferability of identical VAF thresholds.

We acknowledge that the authors appropriately performed 1000 Monte Carlo iterations to mitigate potential bias from threshold selection. However, this correction primarily addresses inflation of statistical significance caused by searching multiple thresholds and does not fully resolve optimism in effect estimates, information loss caused by dichotomizing continuous VAF values, or instability of selected thresholds. These issues represent recognized challenges in developing multivariable prediction models, particularly when sample size is limited relative to model complexity.^4^

Future investigations should consider modeling VAF as a continuous variable or applying prespecified, platform-independent thresholds, followed by validation in independent cohorts before considering incorporation of VAF-based approaches into AML risk stratification frameworks.
